# Author's reply: When does IAPA begin? Interpreting corticosteroid dose thresholds in a target trial emulation

**DOI:** 10.1016/j.aicoj.2026.100149

**Published:** 2026-09-04

**Authors:** Peng Li

**Affiliations:** Department of Infection Control, Henan Provincial People’s Hospital, People’s Hospital of Zhengzhou University, Zhengzhou, China

To the Editor,

We are grateful to Dr. Kocatas for her careful reading of our study and for raising important methodological concerns [1]. We welcome the opportunity to clarify our design and interpretation.

Regarding the timing of IAPA diagnosis, we agree that early-onset cases (median 3 days) might raise concern about prevalent infection at ICU admission. However, our design deliberately used the 7-day pre-ICU period solely as an exposure assessment window to define baseline treatment assignment under the intention-to-treat principle, while ICU admission served as time zero for follow-up. This is a key feature of target trial emulation [2]. By fixing exposure status before time zero rather than at ICU admission, we ensured that the exposure window is completed before followup starts, thereby preserving the correct temporal sequence between exposure assignment and outcome assessment. Consequently, early post-admission diagnoses are much less likely to represent pre-existing disease that would have biased our estimates. Moreover, the frequency of diagnostic procedures was similar between exposure groups (Supplementary Table S4) [3], arguing against surveillance bias that could have led to differential early detection. Given these design safeguards, we did not perform sensitivity analyses that sequentially excluded early-diagnosed cases, and we consider such analyses unnecessary for the validity of our primary findings.

Concerning the competing risk of death, we fully acknowledge that the Cox model estimates cause-specific hazards, and the HR = 1.5 and HR = 2.0 dose points are hazard ratios, not proportional changes in cumulative incidence. We explicitly described these thresholds as derived from the hazard function, which remains a clinically interpretable measure of the relative rate of IAPA occurrence. Our objective was to estimate the effect of pre-ICU corticosteroid dose on the instantaneous risk of IAPA, not to predict absolute risk over time. While we agree that competing events exist, we believe that presenting cause-specific hazard ratios is appropriate for this purpose, and we have been careful in our wording to avoid overinterpreting these values as absolute risk increases.

Finally, regarding the SOFA>7 cutoff, as we clearly stated in both the Discussion and Limitations sections, our subgroup findings are exploratory and hypothesis-generating. We fully agree that independent validation in prospective cohorts is essential before these severity-specific thresholds can be adopted as fixed clinical decision points. We hope our work will stimulate such validation studies.

We thank Dr. Kocatas again for her constructive comments, which help highlight the nuances of our analytical approach and the need for cautious interpretation.

Sincerely,

Peng Li

## Authors' contributions

PL conceptualized the reply, wrote a first draft of the manuscript, revised the text and approved the final draft.

## Consent for publication

Not applicable.

## Ethics approval and consent to participate

The study protocol was approved by the ethics committees of all participating hospitals (lead registry number: HNSRY2023-37). Given the non-interventional, observational design and the use of fully anonymized patient data, the requirement for individual informed consent was waived.

## Funding

Not applicable.

## Availability of data and material

The raw data supporting the conclusions of this article will be made available by the authors, without undue reservation.

## Declaration of competing interest

No competing interests.

## References

[1] Kocataş D.K. (2026). When does iapa begin? Interpreting corticosteroid dose thresholds in a target trial emulation. Ann Intensive Care..

[2] Hernán M.A., Dahabreh I.J., Dickerman B.A., Swanson S.A. (2025). The target trial framework for causal inference from observational data: why and when is it helpful?. Ann Intern Med.

[3] Xu S., Liu H., Fan P., Dou H., Guo L., Qiao B. (2026). Dose-response relationship of pre-ICU corticosteroids and influenza-associated pulmonary aspergillosis: a multicenter target trial emulation. Ann Intensive Care.

